# Improving the Phototherapeutic Efficiencies of Molecular and Nanoscale Materials by Targeting Mitochondria

**DOI:** 10.3390/molecules23113016

**Published:** 2018-11-18

**Authors:** Fengming Lin, Yan-Wen Bao, Fu-Gen Wu

**Affiliations:** State Key Laboratory of Bioelectronics, School of Biological Science and Medical Engineering, Southeast University, Nanjing 210096, China; linfengming@seu.edu.cn (F.L.); byw@seu.edu.cn (Y.-W.B.)

**Keywords:** nanomedicine, cancer therapy, PDT, PTT, subcellular organelle-targeting

## Abstract

Mitochondria-targeted cancer phototherapy (PT), which works by delivering photoresponsive agents specifically to mitochondria, is a powerful strategy to improve the phototherapeutic efficiency of anticancer treatments. Mitochondria play an essential role in cellular apoptosis, and are relevant to the chemoresistance of cancer cells. Furthermore, mitochondria are a major player in many cellular processes and are highly sensitive to hyperthermia and reactive oxygen species. Therefore, mitochondria serve as excellent locations for organelle-targeted phototherapy. In this review, we focus on the recent advances of mitochondria-targeting materials for mitochondria-specific PT. The combination of mitochondria-targeted PT with other anticancer strategies is also summarized. In addition, we discuss both the challenges currently faced by mitochondria-based cancer PT and the promises it holds.

## 1. Introduction

Cancer phototherapy (PT), including photodynamic therapy (PDT) and photothermal therapy (PTT), is regarded as a promising noninvasive method to eradicate tumors with precise spatiotemporal control and reduced side effects. In PT, the administrated photoresponsive agents (including photosensitizers (PSs) and photothermal agents (PTAs)) absorb light to convert oxygen to reactive oxygen species (ROS) in PDT or to generate hyperthermia in PTT, which may eventually cause cancer cell death. Targeted phototherapy at the tissue (primary targeting), cell (secondary targeting), and organelle (tertiary targeting) levels can improve the efficiency of PT, achieve negligible systemic toxicity, and address some significant issues in the field of cancer therapy, like the drug resistance of chemotherapeutics, uncontrollable drug delivery and release, and undesirable damage to normal tissues/cells. Subcellular compartments like the nucleus [1,2,3], mitochondria [4,5], endoplasmic reticulum [6], and plasma membrane [7,8,9] have become targeting sites to enhance anticancer efficiency. Among them, mitochondria, energy producing cellular organelles, are garnering ever-increasing attention because they are crucial to maintain the normal state and function of a cell. It is widely known that the dysfunction of mitochondria may lead to a wide variety of diseases, including cancer, degenerative diseases, diabetes, and aging [10,11,12]. Mitochondria spread wildly in the cytoplasm, and no additional barriers, like karyotheca, need to be overcome for drugs to be delivered to the mitochondria [13]. More importantly, mitochondria are highly sensitive to ROS and heat, presenting excellent locations for organelle-targeted phototherapy. Therefore, the development of mitochondria-targeted PT to fight against cancer is gaining momentum. 

Over the last two decades, mitochondria targeting has been considered an efficient method for different therapeutic purposes as it causes mitochondrial dysfunction. Thus, many agents that target the mitochondria have been developed preclinically and clinically to combat various diseases, providing tremendous sources for mitochondria-oriented PT. As far as we know, up until now, no review paper has summarized the advancements of mitochondria-oriented PT. In this review, we first give an overview of strategies for mitochondria-specific PT against cancer. Next, we summarize the integration of mitochondria-targeted PT with other anticancer strategies and the accompanied advantages brought by mitochondria-anchoring PT. Last, we conclude our review by discussing both the challenges currently faced by mitochondria-based cancer phototherapy and the promises it holds. 

## 2. Strategies for Mitochondria-Targeted PT

### 2.1. Mitochondria-Targeted PT Using Lipophilic Cations

Lipophilic cations penetrate lipid bilayers easily and accumulate inside mitochondria against their concentration gradient through the electrostatic interaction with mitochondria that possess a highly negative inner membrane potential (−150 to −170 mV) [4,14]. Based on this, many lipophilic cations, such as triphenylphosphonium (TPP) cations, cyanine cations, pyridinium, quaternary ammonium salt, isoquinolinium, cyclometalated Ir(III) complexes, and rhodamine derivatives have been used to deliver the bioactive agents of interest to mitochondria for different purposes.

#### 2.1.1. TPP-Based Mitochondria-Targeted PT

TPP is the most frequently explored mitochondria-targeting ligand, both for PT and for other disease therapies, mainly due to its simple structure and easy linkage with target compounds. A number of TPP-conjugated compounds and nanoparticles have been developed for mitochondria-anchoring PT [11,15,16,17,18,19,20]. It has been well demonstrated that TPP is a robust mitochondria-anchoring ligand that can successfully promote a variety of photoresponsive agents to the mitochondria as an enhanced PT against cancer.

Porphyrins possess a highly conjugated, heterocyclic macrocycle and display long-wavelength absorption. They are major components of myoglobin and hemoglobin in the blood and thus, are considered safe. Porphyrins were the first explored PSs and are now the most explored PSs for PDT in the area of oncology [21]. Although cationic porphyrins display high affinity for mitochondria via electrostatic interactions [22], they have still been linked with mitochondria-targeting ligands, such as TPP [18,23,24,25], rhodamine B [24,26], and acridine orange [26], to achieve mitochondria-based PT more effectively. When linked to the TPP moiety, porphyrin derivatives can be localized in the mitochondria, showing an improved photosensitizing effect [18,23,24,25]. Meso-tetraphenylporphyrin was conjugated with TPP at either the para- or meta-positions of its phenyl group, resulting in the derivatives P1 and P2 [23]. Both derivatives were well localized at the mitochondria, as evidenced by confocal fluorescence imaging, showing remarkable phototoxicity toward breast cancer cells MCF-7 with negligible dark toxicity. In particular, P1 exhibits higher cellular uptake and lower dark toxicity than P2, indicating that the rational design of the linking mode between PS and TPP plays an important role in the function of the conjugated compounds. Core-modified porphyrins, like dithiaporphyrin, which are considered to be the second-generation of photosensitizers, have also been delivered to the mitochondria after TPP conjugation [24]. The dithiaporphyrin–TPP conjugate shows high cellular uptake and strong PDT efficacy. Nevertheless, the mitochondrial localization of this conjugate is less efficient than the dithiaporphyrin–rhodamine B conjugate [24].

Metalloporphyrins were initially explored in biology as phosphorescent agents for detecting the oxygen level [27]. They have been applied in PDT, since they are capable of generating ^1^O_2_. After TPP conjugation, platinum(II)–porphyrin can target the mitochondria and lead to the light-induced inhibition of mitochondrial respiration [18]. This repressed mitochondrial respiration leads to a remarkably increased concentration of intramitochondrial oxygen that is advantageous for PDT and contributes to improved PDT [18].

**Figure 1:**
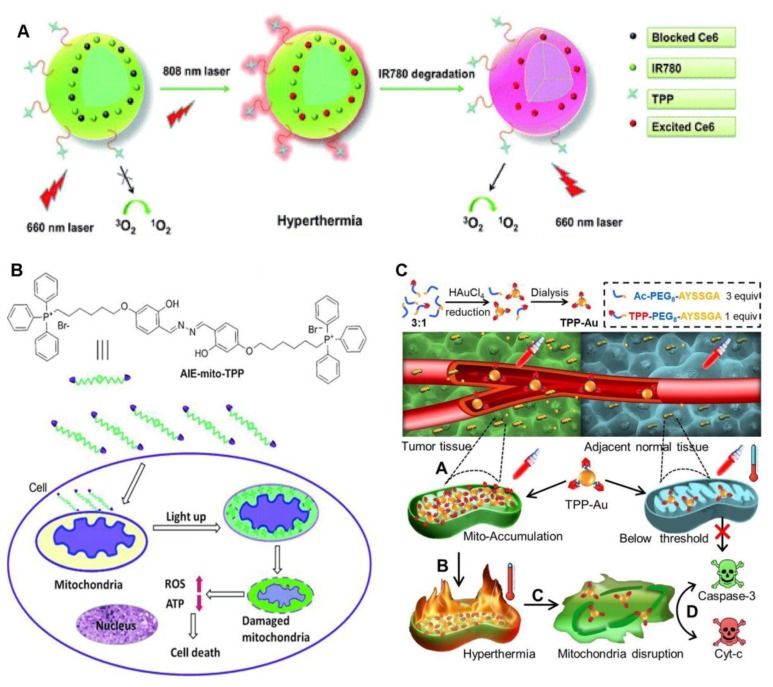
Triphenylphosphonium (TPP)-based mitochondria-targeted phototherapy (PT). (**A**) Schematic of the system TPP-IR780/Ce6. Reprinted with permission from Ref. [29]. Copyright 2016 Royal Society of Chemistry. (**B**) Schematic representation of intracellular tracking and the therapeutic effect of AIE–mito–TPP in cancer cells. Reprinted with permission from Ref. [17]. Copyright 2014 Wiley. (**C**) Mechanism of mitochondria-templated gold nanoparticle accumulation for tumor-selective therapy. Reproduced with permission from Ref. [53]. Copyright 2018 American Chemical Society.

Naturally occurring, chlorin e6 (Ce6), derived from the oxidation of chlorophyll a, is a heterocyclic aromatic molecule with three pyrrole rings and one reduced pyrrole ring. It has a band I absorption maximum of 654 nm. Unfortunately, Ce6 causes long-term skin photosensitization [25]. Even worse, it needs to be administrated at a high dose to be effective [28]. The PDT efficiency of Ce6 can be improved by targeting the mitochondria, presenting a way to reduce the administrated dose of Ce6 [29,30]. Together with the near-infrared (NIR) dye IR780, Ce6 is embedded in TPP-linked theranosomes, achieving mitochondrial accumulation (Figure 1A) and consequently, offering greatly increased PDT efficacy [29]. The presence of IR780 inhibits the phototoxicity of Ce6 through the fluorescence resonance energy transfer between IR780 and Ce6, which can be lifted by the photodegradation of IR780 upon irradiation at 808 nm. In this way, the dark phototoxicity of Ce6 is avoided.

Curcumin, isolated from the spice turmeric, is a yellow dye that serves as a novel, effective PS under blue light [31,32]. It has been reported that curcumin causes mitochondria-mediated apoptosis [33]. Therefore, specific delivery of curcumin-related drugs to the cancer mitochondria may enhance the PDT effect, producing anticancer action. When conjugated with TPP, ternary oxovanadium(IV) complexes of curcumin successfully accumulated in mitochondria and displayed remarkable photocytotoxicity under visible light irradiation through photocleavage of mitochondrial DNA and ROS generation, making most of the treated cancer cells arrest at the sub-G1/G0 phase [34].

Aggregation-induced emission luminogens (AIEgens) with rotor–stator structures are nonluminescent in good solvents, yet become luminescent when they form aggregates that restrict intramolecular motion [35]. AIEgens have the merits of high brightness, a large Stokes shift, and excellent photostability. It has been found that some AIEgens, like tetraphenylethene (TPE)-containing compounds, can effectively generate ROS to efficiently ablate cancer cells [36,37,38]. AIEgens show preferential accumulation in cancer cell mitochondria with the assistance of mitochondria-targeting ligands, like TPP [17,39,40], pyridinium [41,42], isoquinolinium [43,44,45], and cyclometalated Ir(III) complexes [46]. The exploration of AIEgens in mitochondria-targeted phototherapy has had huge success, and several mitochondria-specific AIE PSs have been developed [17,39,41,42,43,44,45,46,47]. For instance, the mitochondria-targeting probe AIE–mito–TPP was designed by linking TPP with a salicyladazine fluoreogen with AIE characteristic (Figure 1B) [17]. This probe could accumulate and turn on fluorescence in mitochondria with more cellular uptake and higher targeting selectivity on cancer cells than on normal ones. Even without light irradiation, AIE–mito–TPP was shown to elevate the ROS level in cancer cells, significantly slash the mitochondrial membrane potential, inhibit ATP production, and affect cancer cell growth, which eventually led to mitochondrial dysfunction and cell death with good selectivity towards cancer cells over normal ones. Two probes—TPECM–1TPP and TPECM–2TPP—containing one and two TPP groups, respectively, have been synthesized with TPECM for both AIE and chemotherapy [39]. The fluorescence of these two compounds was turned on after selective accumulation within cancer mitochondria, resulting in a noticeable enhancement in PDT efficacy through the generation of ^1^O_2_ to induce cancer cell apoptosis. Additionally, TPECM–2TPP posed potent chemo-cytotoxicity on the cancer cells by depolarizing the mitochondrial membrane potential to achieve imaging-guided PDT and chemotherapy. 

Metal complexes show excellent photophysical properties, remarkable photostability, and great solubility in aqueous solutions compared to traditional photoresponsive agents. By linking with TPP, different kinds of metal complexes have been selectively localized to the mitochondria, including iridium(III) complexes [19] and Ru(II) complexes [48,49,50]. In this way, the phototherapeutic efficiencies of these metal complexes were enhanced. Iridium(III) complexes have oxygen-sensitive, long-lived phosphorescence and are considered to be promising PDT agents [19]. After TPP conjugation, these mitochondria-targeted complexes were shown to suppress mitochondrial respiration under hypoxia, resulting in a higher level of intracellular oxygen, which is beneficial for PDT in hypoxic cancer cells. The merits of Ru(II) complexes include fast systemic clearance and practical excitation wavelengths for cells. These emerging PSs have been successfully used in cancer therapy. The TPP-linked Ru(II) complexes mainly accumulate in the mitochondria [48,49,50], triggering cell death by generating notable ^1^O_2_ upon one- and two-photon irradiation [48,49] or by NO release and PTT [50]. Under dark conditions, they are almost nontoxic to cells. 

TPP has been used to localize gold nanostars (AuNSs) [51] and gold nanoparticles [52,53] to produce improved PTT against cancers. AuNSs were comodified via Au–S bonds with the proapoptotic peptide TPP–KLA to target and disrupt mitochondria, and cationic peptide R_8_ to increase the surface zeta potential to form AuNS–pep [51]. AuNS–pep was encapsulated into a hyaluronic acid (HA) protective shell together with the anticancer drug doxorubicin (DOX) through electrostatic interactions, resulting in the nanoplatform AuNS–pep/DOX@HA. This nanoplatform could specifically target cancer cells by CD44 receptor-assisted recognition with HA. Inside the tumor cells, HA was degraded by hyaluronidase, and the nanoplatform was disassembled, releasing AuNS–pep and DOX. The released AuNS–pep mainly accumulated in mitochondria to achieve an improved PTT outcome upon NIR irradiation, while DOX entered the nucleus, achieving enhanced chemotherapy. Interestingly, the cellular DOX retention was prolonged in the presence of AuNS–pep under NIR irradiation, which caused mitochondrial dysfunction and inhibited the energy-related drug efflux pathway. In vivo experiments revealed that AuNS–pep/DOX@HA exhibited a noticeable tumor inhibition capability. On the other hand, gold nanoparticles were modified with TPP moieties to achieve mitochondria-targeted photothermal ablation of cancer [52]. These nanoagents could be further integrated with 3-bromopyruvate, a glycolysis inhibitor, to inhibit both the glycolytic pathway and the mitochondrial oxidative phosphorylation to produce a concerted chemo-photothermal cancer treatment [52]. Sometimes, TPP groups on the surfaces of gold nanoparticles may lead to nonspecific adsorption and aggregation in vivo, as observed with TPP–Au [53]. TPP–Au can be synthesized by the reduction of HAuCl_4_ and TPP–polyethylene glycol (PEG)_8_–AYSSGA, in which the reductive AYSSGA segment also serves as the anchor and linker to mediate the formation of gold nanoparticles (Figure 1C). To solve the instability issue induced by the addition of TPP–PEG_8_–AYSSGA, Ac–PEG_8_–AYSSGA was used to endow TPP–Au with good colloidal stability at an Ac–PEG_8_–AYSSGA to TPP–PEG_8_–AYSSGA molar ratio of 3:1. The as-formed TPP–Au predominantly accumulated in cancer mitochondria, exceeding a concentration threshold to activate an interparticle plasmonic coupling effect that triggered hyperthermia upon NIR irradiation. In this way, the cancer cells were killed with a high selectivity over the adjacent normal cells. This strategy has been demonstrated both in vitro and in vivo. 

Magnetic particles (MPs), like iron oxide particles, are excellent NIR-sensitive PTAs for cancer PTT, since they are nontoxic and compatible with magnetic resonance imaging technology. A novel mitochondria-specific iron oxide NP, Mito-CIO, was synthesized with TPP for mitochondrial targeting, and coumarin was used for fluorescence imaging [54]. Mito-CIO was mainly found in the mitochondria, resulting in better hyperthermia and higher cytotoxicity to HeLa cells upon laser irradiation than to cells treated with coumarin iron oxide (CIO), which was localized in lysosomes. Sometimes, a large number of MPs have been used to achieve high radiation-to-heat conversion, which simultaneously increases the amount of damage done to normal cells. Mitochondria-targeted PTT can enhance the photocytotoxicity for a given number of PTAs by delivering MPs to thermally susceptible mitochondria, reducing the dose of administrated PTAs and therefore, the side effects.

Titanium dioxide (TiO_2_) has a high efficiency, great stability, and is nontoxic. It is thus considered to be an excellent candidate for PSs. Nevertheless, TiO_2_ is activated by ultraviolet (UV) light, which is cytotoxic to living cells, and it has low tissue penetration. Efforts have been devoted to photoactivating TiO_2_ with NIR light, for example, by coating it on the surface of the upconversion nanoparticles [55,56] and by synthesizing a new kind of green titania [57]. Some of these NIR light-irradiated TiO_2_ derivatives have been delivered to mitochondria by linking with TPP [56,57] to improve mitochondria-based PT. For instance, Yu et al. coated a layer of TiO_2_ on the surface of upconversion nanoparticles (UCNPs), leading to the formation of UCNPs@TiO_2_ [56]. Then, TPP was coated on the surface of UCNPs@TiO_2_, resulting in UCNPs@TiO_2_–TPP. UCNPs@TiO_2_–TPP caused an ROS burst in mitochondria, triggering a domino effect. This nanophotosenstitizer displayed excellent mitochondria-targeted PDT performance.

Apart from the examples mentioned above, other PSs, such as methylene blue [58] and coumarin–5-aminolevulinic acid (ALA) [59], and photothermal agents, such as graphene [60] and metal–organic frameworks (MOFs) [61] have been successfully conjugated with TPP to achieve mitochondria-based PT for cancer treatments. 

To sum up, TPP is a powerful and well-explored mitochondria-targeting ligand that can successfully deliver a variety of PSs/PTAs to the mitochondria of cancer cells. Since amine and carboxyl-terminated TPP molecules are commercially available, the TPP ligands can easily be conjugated to the carboxyl/amine-containing phototherapeutic molecular and nanoscale materials via the carbodiimide coupling reaction, which endows these materials with potential mitochondria-targeting capability. In addition, TPP is a low molecular weight molecule and the linkage of TPP to a desired material will not significantly affect the properties and functions of the material. However, the potential toxicity derived from the positive charge and benzene groups of TPP may hinder its wide application in the biomedical field.

#### 2.1.2. Non-TPP Lipophilic Cations for Mitochondria-Oriented PT

Besides TPP, other lipophilic cationic systems like cyanine, pyridinium, quaternary ammonium salt, isoquinolinium, cyclometalated Ir(III) complexes, and rhodamine derivatives have been successfully exploited as mitochondria-targeting ligands.

##### Cyanine Dyes

Cyanine dyes are small, organic, heptamethine dyes that have two nitrogen-containing aromatic heterocyclic rings conjugated by a polymethine bridge. Most cyanine derivatives exhibit strong NIR absorption with high fluorescence quantum yields and molar extinction coefficients. As a result, these dyes have found wide application as probes for fluorescence imaging and PTAs/PSs for PTT/PDT. Among them, indocyanine green (ICG) has been approved by the United States Food and Drug Administration (FDA) for clinical use [62]. Moreover, cyanine dyes are lipophilic and cationic, and some of them can accumulate in the mitochondria. They have been extensively explored to probe aspects of mitochondrial physiology, including mitophagy, membrane potential, pH, polarity, glutathione, cysteine, ROS, and nitroreductase activity [4,63]. Apparently, the heptamethine core of cyanine dyes plays a key role in mitochondrial targeting [63,64,65]. Some cyanine dyes can serve as PSs, which may be related to their molar absorption coefficients, polarizability, and lipo-hydropartition coefficients [66,67]. 

In 2012, Tan et al. reported that the cyanine dye IR-808 was mainly retained in the mitochondria of tumor cells. However, its low ^1^O_2_ generation efficiency limits its application as an efficient PDT [68]. This work showed the potential for using cyanine dyes for mitochondria-targeted PT. In 2013, they modified the lipophilic cationic heptamethine core of cyanine dye with different *N*-alkyl side chains to obtain an NIR small-molecule photosensitizer, IR808DB [69]. The authors declared that IR808DB is more sensitive to light irradiation and more efficient for the PDT of tumors, compared to IR808. Unfortunately, the level of the ^1^O_2_ production was not measured in that work, so it unknown whether the photocytotoxicity was from the PDT. Subsequently, since 2017, several studies have reported cyanine dye-based mitochondria-targeted PDT [70], PTT [71,72,73,74], or combined PDT/PTT [75].

**Figure 2:**
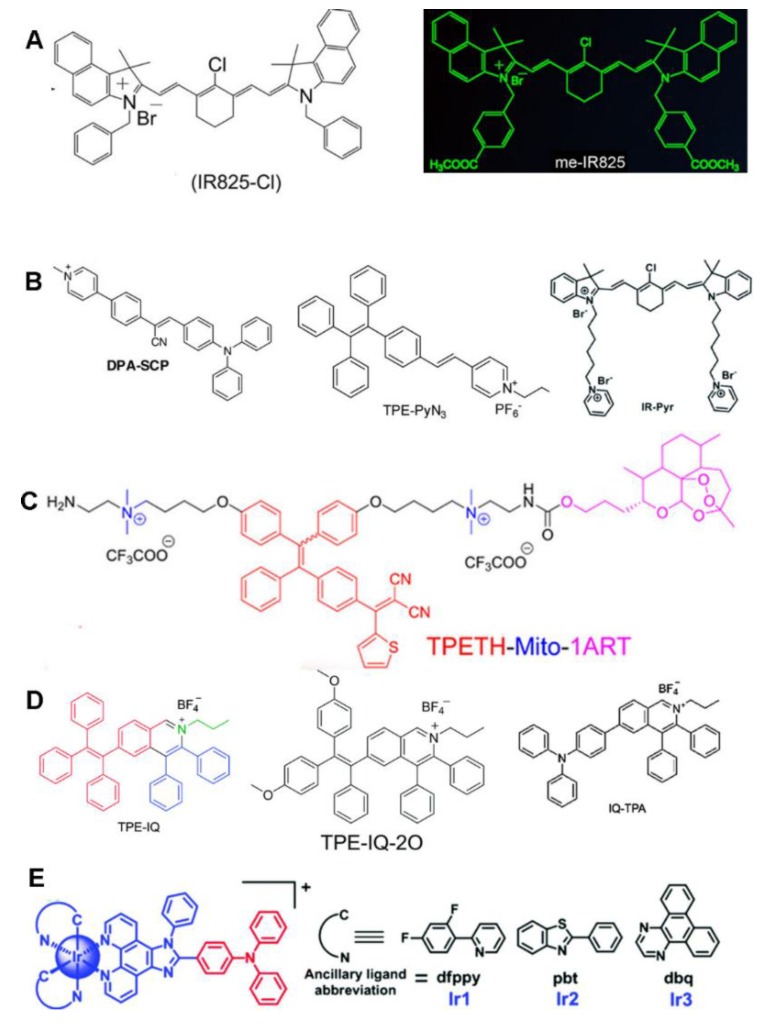
Non-TPP lipophilic cations for mitochondria-specific PT. (**A**) Cyanine dyes. Reprinted with permission from Ref. [72,73]. Copyright 2017 and 2018 American Chemical Society. (**B**) Pyridinium. Reprinted with permission from the Ref. [41,42,70]. Copyright 2017 and 2016 Wiley and Copyright 2017 Royal Society of Chemistry. (**C**) Quaternary ammonium salt. Reproduced from Ref. [47]. Copyright 2018 American Chemical Society. (**D**) Isoquinolinium derivatives. Reproduced from Ref. [43,44,45]. Copyright 2014, 2017, and 2018 Royal Society of Chemistry. (**E**) Cyclometalated Ir(III) complexes. Reproduced with permission from Ref. [46]. Copyright 2017 Royal Society of Chemistry.

In 2017, Jung et al. reported the use of cyanine dyes for mitochondria-targeted PTT [71]. A novel mitochondria-oriented PTA was developed by chemically conjugating cryptocyanine with two TPP moieties, which was named Mito–CCy. As it was designed rationally, Mito–CCy possesses a low fluorescence quantum yield (QY) of <0.01 and a low singlet oxygen QY of <0.02 to maximize the photothermal conversion efficiency. Despite such a low fluorescence QY, Mito–CCy still exhibited fluorescence under confocal microscopy, allowing NIR imaging-guided PTT. Mito–CCy was localized in the mitochondria and generated heat upon NIR light irradiation, leading to endogenous ROS generation, mitochondrial dysfunction, and cell apoptosis. Although most cyanine dyes themselves are preferentially localized in mitochondria, they are sometimes still linked to other well-known mitochondria-targeting moieties, such as pyridinium [70] and TPP [71,76], to target mitochondria more specifically to achieve enhanced PT. Two mitochondria-targeting PTAs derived from cyanine dyes were developed by our group without the introduction of other mitochondria-specific ligands [72,73]. One IR825-Cl (later termed dc-IR825) which includes a lipophilic indolium cation and a benzyl group (Figure 2A) [72]; the other is me-IR825, which has two methyl ester groups that replace the two carboxyl groups of IR825 (Figure 2A) [73]. Both dyes were shown to be rapidly and massively internalized by cancer cells, allowing them to selectively accumulate in mitochondria and achieving effective fluorescence imaging-guided PTT under NIR irradiation. More interestingly, they exhibited red fluorescence with an excitation wavelength of ~550 nm without heat generation, and generated heat under 808 nm laser irradiation. These two dyes offer a dual-channel activatable cancer theranostic, producing fluorescence and heat separately at different wavelengths of light. Further, our group constructed a new PTA by conjugating the hydrophobic amine-containing heptamethine cyanine molecule IR825-NH_2_ with a double hydrophilic block copolymer, methoxypoly(ethylene glycol)_5K_-block-poly(l-aspartic acid sodium salt)_10_ (abbreviated as PEG–PLD), via an amine–carboxyl reaction [74]. The as-designed PEG–PLD(IR825) could form nanomicelles in aqueous solution and achieve highly efficient mitochondria-targeted PTT.

Zinc phthalocyanine (ZnPc) PSs were conjugated with 1-(3-methyl) imidazoliumylethyloxy, resulting in the ZnPc1 complex [77]. ZnPc1 exhibited preferential mitochondrial accumulation when entering the cells. Excellent photocytotoxicity was found for this substance with low dark cytotoxicity. The cancer cell death pathway mainly involved apoptosis, as demonstrated by ROS generation, a diminished mitochondrial membrane potential, and chromatin condensation. In two other reports, ZnPc was integrated with TPP to specifically target mitochondria [78,79]. ZnPc was encapsulated into the mitochondria-oriented polymer PLGA-*b*-PEG–TPP, forming nanoparticles (T–ZnPc–NPs) [78]. Unfortunately, no data were presented to show how well T–ZnPc–NPs localize to mitochondria, although mitochondria-targeted-PDT-activated immunotherapy was observed. Later, Yue et al. used a similar strategy to construct a novel mitochondria-specific drug, ZnPc/CPT–TPPNPs, by encapsulating ZnPc into the block copolymer TL–CPT–PEG_1K_–TPP [79]. ZnPc/CPT–TPPNPs target mitochondria with high specificity, showing elevated PDT efficacy.

##### Pyridinium

Since pyridinium accumulates in the mitochondria when entering the cells, it has been utilized to specifically deliver AIEgens [41,42] and triphenylamines (TPAs) [80] to the mitochondria for PT. Pyridinium has also been incorporated into indocyanine derivatives to improve their properties for mitochondrial localization [70].

The AIEgen DPA–SCP consists of α-cyanostilbene as a building block for AIE, with the diphenylamino (DPA) group serving as a strong electron-donating moiety for red emission and light-induced ^1^O_2_ generation and pyridinium serving as the mitochondria-targeting ligand (Figure 2B) [41]. DPA–SCP is a mitochondria-specific photosensitizer with high light-controlled ^1^O_2_ generation for PDT application. Interestingly, at low concentrations, DPA–SCP increases the ^1^O_2_ level in the mitochondria without damaging the cells upon light irradiation, which improves the sensitivity of cancer cells to ionizing radiation. In another report, TPE–PyN_3_ was prepared through the conjugation of pyridinium units and TPE through vinyl functionality (Figure 2B) [42]. Pyridinium is a mitochondria-targeting ligand, while TPE exhibits AIE properties. TPE–PyN_3_ demonstrates a high affinity for mitochondria and is very sensitive to the mitochondrial membrane potential. Thus, it can monitor cell apoptosis by mitochondrial imaging with high photostability over a long time period. 

TPA derivatives are newly-developed potential PSs for two-photon excited PDTs [80]. When functionalized with cationic moieties, like pyridinium or benzimidazolium, the resulting conjugates are specifically localized in mitochondria and display phototoxic effects upon two-photon NIR irradiation, mediated through the mitochondrial apoptotic pathway via ROS production. As a new kind of water-soluble organic PS, these TPA cationic derivatives hold great promise for in vivo PDT given their mitochondrial localization and large two-photon absorption cross-sections from 760 to 860 nm. 

An indocyanine derivative was developed by the incorporation of a pyridinium ion into the indocyanine skeleton (Figure 2B) [70]. This compound exhibits excellent water solubility and increased stability under both light and dark conditions in comparison with IR-780. With the combination of hyaluronic acid, it forms micelles that can selectively bind to cancer mitochondria to achieve enhanced PDT efficacy.

##### Quaternary Ammonium Salt

Lipophilic quaternary ammonium salts have been chemically conjugated with an AIEgen tetraphenylethenethiophene (TPETH) (Figure 2C) [47] and a novel NIR photosensitizer [81] to specifically deliver the formed conjugates to the mitochondria for cancer PT. The probe TPETH-Mito-1ART possesses a TPETH core as the AIE-active fluorophore and PS, two quaternary ammonium salt arms for cancer mitochondrial localization, and artemisinin (ART) for chemotherapy on one arm [47]. TPETH-Mito-1ART has been shown to predominantly accumulate and light up in the mitochondria of cancer cells, but has difficulty penetrating the normal cells. ART was activated in the mitochondria to impose chemotherapy to cancer cells, while TPETH generated plenty of toxic ROS that damaged the mitochondria and promoted the therapeutic effect further.

Recently, Liu et al. achieved NIR fluorescence imaging-guided chemo-photodynamic therapy in a single molecule by fabricating a mitochondria-oriented prodrug, PNPS [81]. PNPS contains two components: a novel quaternary ammonium salt structured NIR photosensitizer (NPS) for mitochondria-targeted PDT and an anticancer drug, 5′-deoxy-5-fluorouridine, for chemotherapy. These two components are linked through a bisboronate group that can be broken in the presence of H_2_O_2_. Hence, in cancer cells with a high H_2_O_2_ concentration, PNPS releases free NPS and free 5′-deoxy-5-fluorouridine, allowing effective PDT and chemotherapy treatments, respectively. 

##### Isoquinolinium

Although instances of research on the mitochondrial targeting ability of isoquinolinium molecules are rare [82], they can successfully deliver AIEgens to mitochondria to achieve imaging-guided PT (Figure 2D) [43,44,45]. An isoquinolinium-derived compound, TPE–IQ, was synthesized [43]. TPE–IQ achieved mitochondria-specific imaging and produced ROS with UV irradiation which led to effective cell apoptosis. The incubation and irradiation time required for TPE–IQ was short, even at a low working concentration. Therefore, TPE–IQ is a promising theranostic agent. Nevertheless, the short excitation wavelength in the UV region may give rise to side effects to normal tissues if a long exposure time is needed. To solve this issue, the molecular structure of TPE–IQ was modified with the addition of two electron-donating methoxy groups, resulting in positively-charged AIEgen TPE–IQ–2O [44]. TPE–IQ–2O was shown to localize to the cancer mitochondria with superb selectivity and could distinguish cancer cells from normal ones. Upon white light irradiation, it effectively generated ROS to induce cancer cell death. Hence, TPE–IQ–2O represents a promising theranostic AIEgen for PDT by targeting mitochondria. Currently, most AIE-based imaging-guided PDT treatments use white light, the single-photon excitation, for irradiation [17,39,41,47]. This conventional single-photon excited PDT cannot achieve precise three-dimensional treatment and deep tissue penetration which impedes the practical application of PDT. In this context, two-photon excited PDT (TP–PDT) is a good choice. For imaging-guided TP–PDTT, the moiety of TPE–IQ was replaced with a triphenylamine (TPA) group to produce IQ–TPA [45]. TPA can serve as both an AIEgen and a potential two-photon absorption cross-section enhancer. IQ–TPA was shown to have a high two-photon absorption cross-section of 213 M, noticeable ROS production, and mitochondria-anchoring ability, all endowing it with great feasibility for imaging-guided TP–PDT.

##### Cyclometalated Ir(III) Complexes

In recent years, increasing attention has been paid to the development of cyclometalated Ir(III) complexes as anticancer drugs and/or PSs [83,84]. Cyclometalated Ir(III) complexes show a prominent affinity for mitochondria, probably via the action mechanisms of lipophilic cations [85]. AIEgen TPA was linked to cyclometalated Ir(III) complexes to achieve similarly AIE-active TP–PDT in the mitochondria (Figure 2E) [46]. These AIE-active Ir(III) complexes were shown to selectively localize in the mitochondria with aggregation-triggered PDT activity and display more preferential photocytotoxicity towards cancer cells than normal ones. 

##### Rhodamine Derivatives

Thanks to their fast equilibration and intrinsic fluorescence, the rhodamine-based lipophilic cation is one of the well-known probes for the mitochondrial membrane potential of cultured cells [86]. Rhodamine B, a red fluorescent probe, has excellent photophysical properties and is well-known for its specific mitochondrial targeting ability [86]. By covalent conjugation to rhodamine B, both porphyrin [26] and core-modified porphyrin [24] were shown to be selectively delivered to the mitochondria, showing higher cellular uptake and better phototoxicity than that of free core-modified porphyrin. Rhodamine 123 (Rhod123) is a well-explored commercial dye that specifically images mitochondria. Rhod123 was combined with polydopamine nanoparticles (PDA NPs) together with doxorubicin (DOX), resulting in the formation of PDA–Rhod–DOX [87]. PDA–Rhod–DOX was shown to localize to the mitochondria with high selectivity which reduced ATP generation and caused apoptosis in cancer cells, leading to a high photothermal/chemotherapy synergetic efficiency. 

##### Acridine Orange (AO)

Porphyrin–AO was obtained by chemically conjugating porphyrin with AO through a saturated hydrocarbon linker [26]. Porphyrin–AO has a quaternary amine, similar to nonyl acridine organe that has been demonstrated to preferentially accumulate in the mitochondria. As a result, porphyrin–AO also has the capability to accumulate selectively in mitochondria [26]. However, porphyrin–AO can target not only mitochondria but also nuclei, although it displays substantial intracellular accumulation. Porphyrin–AO has high phototoxicity, but its dark toxicity cannot be ignored. Moreover, porphyrin–AO is not stable in aqueous solution and easily forms aggregates. 

Here, the successful applications of other lipophilic cationic molecules in mitochondria-based PT have been demonstrated. These non-TPP lipophilic cationic molecules, including cyanine, pyridinium, quaternary ammonium salt, and rhodamine, have been less explored than TPP. However, these lipophilic cations share a similar cytotoxicity issue when they accumulate in the mitochondria. Since mitochondria-targeted PT using these lipophilic cations is a common and potent strategy to enhance the phototherapeutic efficiencies of PSs/PTAs, it is definitely worth evaluating the potential toxicity of these molecules in the future.

### 2.2. Mitochondria-Targeted PT Using Peptides

Mitochondria-targeted peptides (MTPs), a potent alternative to lipophilic cations for the localization of bioactive agents to mitochondria, are obtained from nature or by chemical synthesis. MTPs can easily be linked to the cargoes of interest via covalent bonding to achieve mitochondrial targeting for a large number of applications. Studies have been carried out to figure out the structural requirements for a peptide to efficiently target mitochondria, including charge, hydrophobicity, and amino acid sequence. Nevertheless, how MTPs target mitochondria is still unknown. Similar to lipophilic cations, synthetic MTPs are designed to contain hydrophobic amino acids (isoleucine, phenyl alanine, and tyrosine) to penetrate the lipid membrane, positively charged amino acids (lysine and arginine) to bind to the plasma and mitochondrial membranes that are negatively charged through electrostatic interaction. Up until now, the naturally occurring MTPs have generally included mitochondrial localization sequences (MLSs) [88,89,90], the partial sequence of the antibiotic gramicidin S [91], and the peptide sequence CGKRK [92]. Although MTPs have been widely applied to delivery various therapeutic agents, their use in mitochondria-oriented PT is rare and remains largely unexplored. As far as we know, only several studies have investigated peptide-based mitochondria-oriented PT [93,94,95,96].

MLSs generally consist of 20 to 30 amino acids and target mitochondria via the translocases of the outer and inner membrane complexes of mitochondria [97,98]. Several MLSs have been exploited to deliver drugs/molecules to the mitochondria [88,89,90]. Nevertheless, MTPs may be degraded in vivo by proteinases, which can be overcome by the incorporation of the D-isomer of arginine [99]. Using a PEG spacer, porphyrin was attached to an MLS, MSVLTPLLLRGLTGSARRLPVPRAKIHSL, that was obtained from the subunit VII of human cytochrome c (Cyt c) oxidase [100], leading to significant cellular accumulation [93]. Unfortunately, this porphyrin–MLS conjugate has been shown to accumulate mainly in lysosomes rather than in mitochondria. When trapped in lysosomes, the porphyrin–MLS conjugate loses its MLS through peptide degradation by lysosomal enzymes and fails to target mitochondria [93]. Unsuccessful mitochondrial targeting has also been found in other porphyrin–peptide conjugates [101,102,103]. 

**Figure 3:**
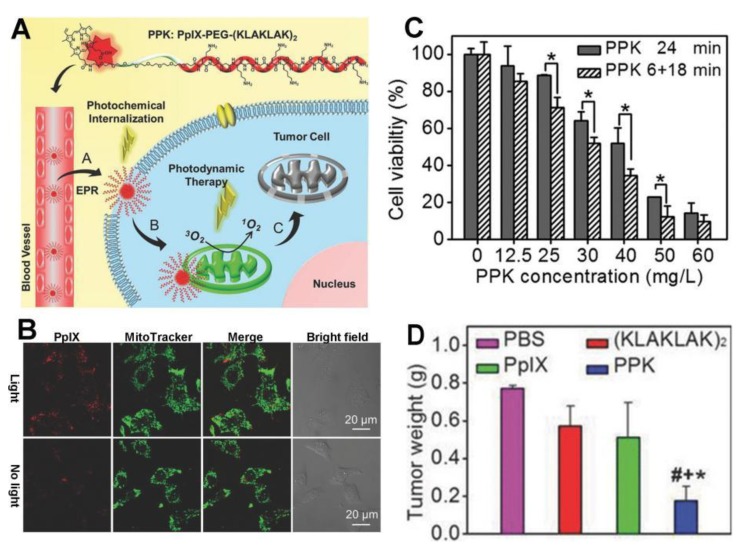
(**A**) Peptide-based mitochondria-targeted PT. Peptide (KLAKLAK)_2_ was linked with the photosensitizer (PS) protoporphyrin (PpIX) by a short PEG linker to achieve mitochondria-oriented photodynamic therapy (PDT). (**B**) Fluorescence imaging of HeLa cells treated with PPK (PpIX–PEG–(KLAKLAK)_2_) with and without light. (**C**) Cell viability of HeLa cells in the presence of PPK under 24 min and 6 + 18 min of light irradiation. * *p* < 0.01, when the group was compared with cells treated with PPK upon 24-min light irradiation as measured by a Student’s *t*-test. (**D**) The average tumor weight at day 12 after treatment. ^#^ *p* < 0.05, ^+^ *p* < 0.05, and * *p* < 0.05 were determined by a Student’s *t*-test when the group was in comparison with the groups that were treated with PBS, (KLAKLAK)_2_, and PpIX, respectively. Reprinted with permission from Ref. [94]. Copyright 2015 Wiley.

α-Helical amphipathic peptide (KLAKLAK)_2_ was initially designed as a synthetic antibacterial peptide. This peptide damages the mitochondrial membrane and causes apoptotic cell death. The KLAKLAK-based strategy has been successfully applied to specifically deliver the photosensitizer protoporphyrin (PpIX) to the mitochondria. The self-delivery system PpIX–PEG–(KLAKLAK)_2_, known as PPK, includes PpIX, the amphipathic (KLAKLAK)_2_ peptide for mitochondrial targeting, and a PEG linker between PpIX and the peptide (Figure 3) [94]. PPK has a high drug loading efficiency. The cellular uptake of PPK is improved by the photochemical internalization effect caused by PpIX upon short-term light irradiation. Inside the cells, PPK specifically targets the mitochondria and generates plenty of ROS under another long period of light irradiation, showing greatly enhanced PDT efficiency. As demonstrated using a murine model, this PPK-based, dual-stage, light-controlled self-delivery system exhibits effective long-time tumor inhibition with long tumor retention and negligible systemic toxicity. In another study, Han et al. developed an amphiphilic chimeric peptide, PpIX–6-aminohexanoic acid–PEG_8_−d(KLAKLAK)_2_–GRGD (PpIX–Ahx–PEG_8_−d(KLAKLAK)_2_–GRGD), which is also known as MTCP [95]. It contains PpIX for PDT and proapoptosis-inducing peptide (KLAKLAK)_2_ for mitochondrial targeting. MTCP can encapsulate the anticancer drug DOX with high efficiency, forming nanoparticles in solution. With an enhanced cellular internalization, the resulting MTCP/DOX can be preferentially located in the mitochondria, leading to sustained release of DOX and in situ ^1^O_2_ generation to disrupt the mitochondria. Therefore, combined chemo-phototherapy is achieved. Meanwhile, the intracellular ATP is dramatically reduced, which suppresses the efflux of DOX mediated by ATP-dependent efflux transporters remarkably.

To date, the mechanism behind the mitochondria-targeting of peptides remains unclear. Unlike lipophilic cations, peptides are degradable and may not induce severe cytotoxicity after mitochondrial accumulation. However, it was reported that the peptide degradation may cause failed mitochondria-targeting in some cases [93]. Additionally, peptides may induce undesired immune responses, and the synthesis of some peptides is not that easy. Moreover, some peptides have large molecular weights, which may lead to difficulty in their successful conjugation with target materials and/or may affect the properties and functions of the target materials.

### 2.3. Mitocondria-Targeted PT Based on Aptamers

Since Cyt c is normally bound to the inner mitochondrial membrane by the anionic phospholipid cardiolipin, the Cyt c-specific binding aptamers have been conjugated with functional nanoparticles for cancer therapy [104,105]. For example, for the first time, Qu’s group reported a mitochondria-targeted drug carrier based on aptamer-conjugated mesoporous silica-encapsulated gold nanorods, which has the ability to load various hydrophobic therapeutic agents acting on mitochondria to enhance the therapeutic efficiency [105]. Mitochondria-targeting using aptamers is a novel and promising platform for mitochondria-based PT. Compared to peptides, aptamers are nonimmunogenic and are easy to synthesize. Moreover, aptamers can resist biodegradation and denaturation by further modifications. However, the relatively large molecular weights of aptamers may lead to a low conjugation efficiency with a target material and exert a substantial influence on the structure and properties of the material. In addition, the high production cost of aptamers may further prevent the clinical translation of aptamer-based and mitochondria-oriented phototherapy.

### 2.4. Nanoparticles with Intrinsic Mitochondrial Targeting Capability

**Figure 4:**
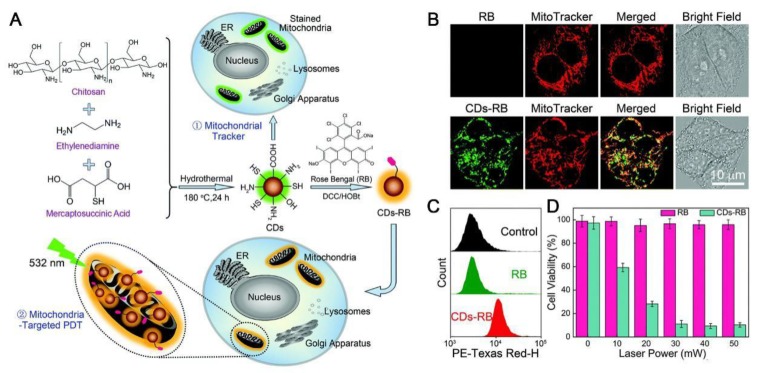
(**A**) Schematic illustrating the synthetic route of CDs and their applications as fluorescent mitochondrial trackers and mitochondria-targeting drug carriers (nanomissiles). (**B**) Confocal images of MCF-7 cells costained with MitoTracker (Ex: 638 nm) and free rose bengal (RB) (Ex: 552 nm) or costained with MitoTracker (Ex: 638 nm) and carbon dots (CDs)–RB (Ex: 488 nm). (**C**) Flow cytometric results of MCF-7 cells incubated without (control) and with free RB or CDs–RB. (**D**) The cell viability result of MCF-7 cells incubated with free RB or CDs–RB for 30 min, followed by washing with PBS twice and then irradiation using a 532 nm laser of different power intensities for 5 min. In cell experiments, free RB and CDs–RB were used at the same RB concentration (5 μg mL^−1^). Reproduced with permission from Ref. [111]. Copyright 2017 Royal Society of Chemistry.

In addition to the strategies of linkage to lipophilic cations or MTPs for mitochondrial targeting, some nanomaterials, such as carbon quantum dots (or carbon dots, CDs), single-walled carbon nanotubes (SWNTs), and graphene oxide (GO), have been reported to be able to selectively target mitochondria [5,106,107,108], possibly via electrostatic interactions between the nanoparticles and the mitochondria. Based on their intrinsic mitochondrial targeting capability, these nanoparticles can be used as mitochondria-targeted theranostic agents without the introduction of extra mitochondriotropic ligands. On the one hand, owing to the presence of various functional groups on the surface, CDs can be easily modified by theranostic drugs for various biomedical applications [2,109,110]. Among the studies of CDs, Hua et al. reported a novel type of fluorescent CDs with intrinsic mitochondrial targeting ability, which was produced by one-step hydrothermal treatment of chitosan, ethylenediamine, and mercaptosuccinic acid (Figure 4) [111]. After conjugation with a photosensitizer rose bengal (RB), the resultant CDs–RB nanomissiles were shown to achieve efficient cellular uptake and mitochondrial targeting/accumulation, achieving mitochondria-targeted PDT. On the other hand, due to their excellent photothermal transducer properties, the mitochondrial SWNTs themselves can be used as mitochondria-targeting photothermal conversion probes [112]. It was observed that SWNTs can be more efficiently accumulated in the mitochondria of cancer cells and can selectively destroy cancer cell mitochondria under NIR laser irradiation. In addition, in our previous work on the subcellular localization of nanoparticles, we found that some nanoparticles can be localized to multiple organelles, including the mitochondria, endoplasmic reticulum, lysosomes, and the Golgi apparatus, enhancing the efficiency of phototherapy [113,114]. These nanoparticles with their intrinsic mitochondrial targeting capability are newly minted materials for mitochondrial orientation. Nevertheless, more effort is required to explore both the mechanisms behind their mitochondrial targeting and the applications of these nanomaterials to deliver various PSs and PTAs to the mitochondria for mitochondria-based PT. 

To summarize Section 2, the various strategies for mitochondria-targeted PT are listed in Table 1.

## 3. Combination of Mitochondria-Targeted PT with Other Cancer Treatment Modalities

### 3.1. Integration of Mitochondria-Targeted PT with Chemotherapy to Overcome Drug Resistance

Although cancer diagnosis and chemotherapy have advanced greatly over the past decades, they still have some drawbacks to overcome, such as drug resistance, side effects, and high relapse rates, highlighting the urgency to investigate novel anticancer strategies. The combination of mitochondria-targeted PT with chemotherapy offers improved specificity of target delivery, relieved drug resistance, reduced side effects, and enhanced therapeutic efficiency by administrating a PS and a chemotherapeutic agent sequentially or simultaneously. For concurrent PT and chemotherapy, the PSs and chemotherapeutic agents are either chemically conjugated or physically encapsulated into the nanocarriers. 

**Figure 5:**
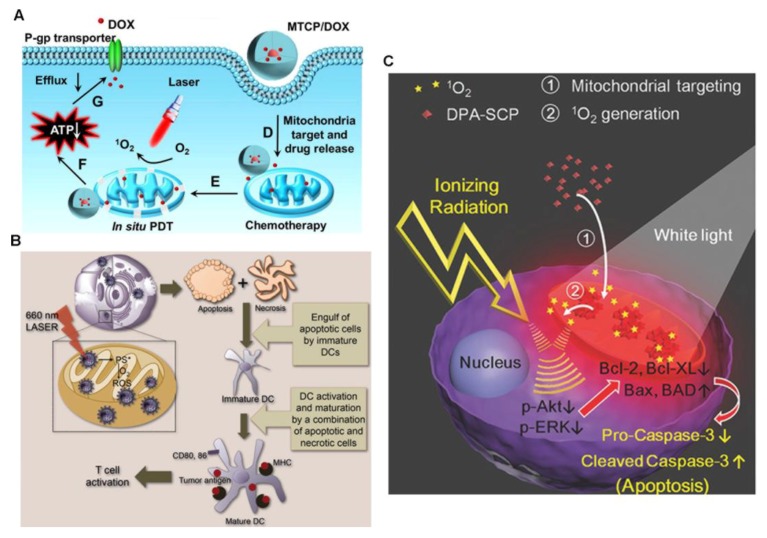
The combination of mitochondria-oriented PT with chemotherapy (**A**), reprinted with permission from Ref. [95]. Copyright 2016 American Chemical Society. The combination of mitochondria-oriented PT with immunotherapy (**B**), reproduced with permission from the Ref. [78]. Copyright 2013 American Chemical Society. The combination of mitochondria-oriented PT with radiotherapy (**C**), reprinted with permission from Ref. [41]. Copyright 2017 Wiley.

The integration of mitochondria-targeted PT with chemotherapy provides a novel strategy to overcome drug resistance, a huge challenge for conventional chemotherapy [95]. The ATP-binding cassette (ABC) transporters play an important role in this complex phenomenon by actively pumping hydrophobic drugs out of cells in an ATP-dependent way that results in decreased intracellular drug concentrations. Even worse, tumor cells express more ABC transporters than normal cells [95], resulting in an increased difficulty for intracellular drug accumulation. Mitochondria are the powerhouses of cells, providing the major energy source for cancer cell proliferation, invasion, and metastasis. Their dysfunction in cancer cells inhibits cellular ATP production. Mitochondria-targeted PT can effectively damage the mitochondria, giving rise to mitochondrial dysfunction and dramatically reducing the intracellular concentration of ATP (Figure 5A) [17,51,95,115]. Without ATP, the efflux of DOX mediated by ATP-dependent efflux transporters can be remarkably suppressed and the retention of chemotherapeutic agents in cancer cells is prolonged, relieving drug resistance. 

### 3.2. Mitochondria-Targeted PT Can Incur Systemic Anticancer Immune Response for Immunotherapy

Tumor cells damaged by PDT release tumor-related antigens that are taken, processed, and presented by antigen-presenting cells, such as dendritic cells, to activate cytotoxic T lymphocytes, enabling the enhancement of the systemic anticancer immune response. These cytotoxic T lymphocytes can move to and kill nonirradiated distant tumors by cellular immunity. It has been reported that the mitochondrial localization of PSs can enhance the host immune response under light irradiation. In a previous study, ZnPc was integrated with TPP to specifically target mitochondria [78]. Upon light irradiation, this mitochondria-targeted compound was shown to produce cancer cell antigens that activate dendritic cells to produce a large amount of interferon-gamma (Figure 5B). Interferon-gamma is a product of T cells and natural killer cells that are involved in the defense against cancer cells [78,116]. When integrated with check-point-blockade therapy using programmed death-ligand 1, the infiltration of cytotoxic T lymphocytes promoted by mitochondria-targeted PDT into distant tumors is greatly enhanced, thus triggering a systemic immune response to erase nonirradiated tumors that are 1 to 2 cm away from irradiated ones [117]. This approach may have the potential to treat metastatic cancers in the future.

### 3.3. Combination of Mitochondria-Targeted PT with Radiotherapy Presents a Novel Strategy to Address Radiation Resistance

Radiotherapy works at the front line to kill cancers such as esophageal, colorectal, and lung cancers. In particular, it is the mainstream treatment to effectively control local cancers and to shrink large tumors that are inoperable so that they are ready for surgery. Radiotherapy often fails in the clinic, mostly due to the development of tumor cell resistance to radiation. To solve this issue, radiosensitizers are utilized to make tumor cells more sensitive to radiotherapy. The effect of this is evaluated by SER10, the sensitizer enhancement ratio at 10% cell survival. Right now, the most popular radiosensitizers are chemotherapeutics like cisplatin and paclitaxel, and gold nanoparticles, which usually have SER10 values of no more than 1.41 [118,119]. Therefore, the development of novel radiosensitizers with larger SER10 values is highly desirable. Interestingly, Yu and coworkers discovered that mitochondria-targeted PDT presents a novel, effective way to sensitize cancer cells to radiation with a high SER10 of 1.62 (Figure 5C) [41]. Their work provided new materials and insights into radiation therapy and demonstrated that the integration of mitochondria-targeted PT with radiotherapy may serve as an excellent strategy to overcome the radiation resistance of cancer cells. 

### 3.4. Incorporation of Mitochondria-Targeted PT with Imaging for Cancer Theranostics

The incorporation of imaging into PT, known as imaging-guided PT, is of great value in fundamental and clinical research, as it simultaneously achieves target detection, drug distribution tracking, and therapeutic effect evaluation in one system. Cancer cells generally have a higher negative mitochondrial potential than normal cells, making mitochondria-anchoring agents accumulate more in cancer cell mitochondria than in normal cell mitochondria to selectively image and/or kill cancer cells over normal cells, which is a great advantage of mitochondria-targeted imaging-guided PT. AIEgens and cyanine dyes are good choices for use in imaging-guided PT with the assistance of mitochondria-targeting ligands. Both AIEgens and cyanine dyes serve as fluorescent therapeutic PSs that fluoresce by themselves upon delivery to mitochondria and thus do not require additional fluorescence tags for imaging, which is highly desired because the addition of a fluorescent tag to a PS may change the cellular fate of the PS and the fluorescence of the tag cannot reflect the position of the drug when it is released. 

The fluorescence of traditional PSs can be quenched at high concentrations or in an aggregated state, which is called aggregation-caused quenching (ACQ). The ACQ of the traditional PSs not only reduces their fluorescence intensity but also decreases their phototherapeutic efficiencies. To resolve this issue, low concentrations of PSs are usually utilized. However, such low loading of PSs leads to easy photobleaching upon laser irradiation, losing both the imaging and PT functions. Alternatively, AIEgens provide a good solution to this ACQ problem. Unlike conventional PSs, AIEgens aggregate and fluoresce. Mitochondria-anchoring AIEgens selectively accumulate in mitochondria and light up upon aggregation, inducing cell stress and causing mitochondrial dysfunction for imaging-guided cancer therapy [17,39,41,42,43,44,45,46,47].

It is well-known that the NIR region (>700 nm) is excellent for bioimaging due to its deep tissue penetration and low tissue fluorescence. The advantages of NIR imaging-guided PT include high sensitivity, real-time imaging, noninvasiveness, and non-radioactivity. Until now, several NIR PSs have been developed to target the mitochondria [72,73,81]. Some cyanine derivatives have strong NIR absorption and have found wide applications in both PTT and fluorescence imaging [72,73].

## 4. Conclusions

In summary, lipophilic cations and peptides are well-known mitochondrial ligands that can successfully deliver different PSs and PTAs to the mitochondria for cancer treatments with different purposes. Although lipophilic cations do not damage mitochondria in vitro, their accumulation in mitochondria results in potential cytotoxicity. In addition, the modification of target molecules and nanomaterials with lipophilic cations, such as TPP, can cause these reagents to become positively charged, which can reduce both their blood circulation time and tumor accumulation. On the other hand, peptides might be degraded in vivo by proteinases, leading to failed mitochondria-targeted PT [99]. Therefore, it is highly desirable to explore the use of other existing mitochondrial targeting ligands and to develop new mitochondria-targetable molecules to achieve mitochondria-targeted PT. Also, mitochondria-targeted PT has been combined with other cancer therapies, including chemotherapy, radiotherapy, and immunotherapy, to form various cancer treatment strategies that have shown enhanced therapeutic efficiencies with less side effects and relieved drug resistance. 

Besides the overcoming of drug resistance, as we mentioned above, mitochondria-targeted PT is also a good method to address the issue of hypoxia-caused PDT inefficiency. Many kinds of solid tumors have insufficient oxygen supply, which is called hypoxia. Tumor hypoxia is a major obstacle for successful cancer therapies like radiotherapy, chemotherapy, and PT. In particular, cancer hypoxia strongly limits the therapeutic efficiency of PDT, which is oxygen-dependent. Mitochondria-targeted PDT has been demonstrated to be an innovative way to overcome this issue [18,19]. 

The use mitochondria-oriented PT has several advantages as a cancer treatment modality: (1) high cancer selectivity due to the higher mitochondrial membrane potential and increased oxidative stress in cancer mitochondria than in normal cell mitochondria; (2) increased local concentration of PSs and PTAs, contributing to lower administrated drug doses and fewer side effects, together with greater cancer selectivity; (3) relieved drug resistance; (4) the overcoming of hypoxia-induced inefficiency of cancer treatment; (5) a high radiosensitization effect in radiotherapy; and (6) an enhanced immune response. Nevertheless, mitochondria-specific PT still have some challenges to overcome, such as low tissue penetration and hypoxia-caused PDT inefficiency. It is believed that mitochondria-targeted PT holds great promise for clinical use to combat cancer.

## Figures and Tables

**Table 1:** Strategies for mitochondria-targeted PT.

Mitochondria-Targeting Ligands	PSs/PTA	Mechanism	Advantages	Disadvantages
Lipophilic cations				
TPP	Porphyrin [18,23,29], curcumin [34], AIEgens [17,39,40], metal complexes [19,48,49,50], metal nanoparticles [51,52,53], methylene blue [58], aminolevulinic acid (ALA) [59], graphene [60], metal–organic frameworks (MOFs) [61]	Lipophilic cations can penetrate lipid bilayers easily and accumulate inside the mitochondria against their concentration gradient through electrostatic interactions with mitochondria that possess highly negative inner membrane potential (–150 to –170 mV) [4,14]	Ease of modification, commercial availability, negligible effect on conjugation	Potential cytotoxicity in vitro and in vivo
Cyanine dyes	Serving as PSs/PTAs themselves [72,73]			
Pyridinium	AIEgens [41,42], triphenylamines [80], indocyanine derivatives [70]			
Quaternary ammonium salt	AIEgen [47], a novel NIR photosensitizer (NPS) [81]			
Isoquinolinium	AIEgens [43,44,45]			
Cyclometalated Ir(III) complexes	AIEgens [46]			
Rhodamine derivatives	Porphyrin [24,26]			
Acridine orange	Porphyrin [26]			
Peptides				
Synthetic peptide	Porphyrin [93]	The same as lipophilic cations	Biodegradability	Failure in targeting due to degradability, undesired immune response, and complicated synthesis
Mitochondrial localization sequences (MLSs)	Porphyrin [94,95]	Via the translocases of the outer and inner membrane complexes of the mitochondria		
Aptamers				
Aptamers	Gold nanorods [105]	The cytochrome C-specific aptamer binds to cytochrome C which resides on the inner mitochondrial membrane by binding to the anionic phospholipid cardiolipin	Non-immunogenicity, easy synthesis, resistance to biodegradation and denaturation	Low conjugation efficiency, high cost
Nanoparticles				
Carbon dots	Rose bengal [111]	Possibly via the electrostatic interactions between the nanoparticles and the mitochondria	No requirement of mitochondrial targeting ligands, capability for use as drug carriers	Large size (not suitable for use as a small label)
Single-walled carbon nanotubes	Serving as PTAs themselves [112]			

## References

[1] Zhu Y.X., Jia H.R., Pan G.Y., Ulrich N.W., Chen Z., Wu F.G. (2018). Development of a light-controlled nanoplatform for direct nuclear delivery of molecular and nanoscale materials. J. Am. Chem. Soc..

[2] Hua X.W., Bao Y.W., Wu F.G. (2018). Fluorescent carbon quantum dots with intrinsic nucleolus-targeting capability for nucleolus imaging and enhanced cytosolic and nuclear drug delivery. ACS Appl. Mater. Inter..

[3] Pan L.M., Liu J.N., Shi J.L. (2018). Cancer cell nucleus-targeting nanocomposites for advanced tumor therapeutics. Chem. Soc. Rev..

[4] Zielonka J., Joseph J., Sikora A., Hardy M., Ouari O., Vasquez-Vivar J., Cheng G., Lopez M., Kalyanaraman B. (2017). Mitochondria-targeted triphenylphosphonium-based compounds: Syntheses, mechanisms of action, and therapeutic and diagnostic applications. Chem. Rev..

[5] Gao G., Jiang Y.W., Yang J.J., Wu F.G. (2017). Mitochondria-targetable carbon quantum dots for differentiating cancerous cells from normal cells. Nanoscale.

[6] Martinon F. (2012). Targeting endoplasmic reticulum signaling pathways in cancer. Acta Oncol..

[7] Jia H.R., Jiang Y.W., Zhu Y.X., Li Y.H., Wang H.Y., Han X.F., Yu Z.W., Gu N., Liu P.D., Chen Z., Wu F.G. (2017). Plasma membrane activatable polymeric nanotheranostics with self-enhanced light-triggered photosensitizer cellular influx for photodynamic cancer therapy. J. Control. Release.

[8] Jia H.R., Zhu Y.X., Xu K.F., Liu X.Y., Wu F.G. (2018). Plasma membrane-anchorable photosensitizing nanomicelles for lipid raft-responsive and light-controllable intracellular drug delivery. J. Control. Release.

[9] Li S.Y., Qiu W.X., Cheng H., Gao F., Cao F.Y., Zhang X.Z. (2017). A versatile plasma membrane engineered cell vehicle for contact-cell-enhanced photodynamic therapy. Adv. Funct. Mater..

[10] Schapira A.H.V., Olanow C.W., Greenamyre J.T., Bezard E. (2014). Slowing of neurodegeneration in Parkinson’s disease and Huntington’s disease: Future therapeutic perspectives. Lancet.

[11] Kwon H.J., Cha M.Y., Kim D., Kim D.K., Soh M., Shin K., Hyeon T., Mook-Jung I. (2016). Mitochondria-targeting ceria nanoparticles as antioxidants for Alzheimer’s disease. ACS Nano.

[12] Balaban R.S., Nemoto S., Finkel T. (2005). Mitochondria, oxidants, and aging. Cell.

[13] Han K., Ma Z.Y., Han H.Y. (2018). Functional peptide-based nanoparticles for photodynamic therapy. J. Mater. Chem. B.

[14] Smith R.A., Porteous C.M., Gane A.M., Murphy M.P. (2003). Delivery of bioactive molecules to mitochondria in vivo. Proc. Natl. Acad. Sci. USA.

[15] Biswas S., Dodwadkar N.S., Deshpande P.P., Torchilin V.P. (2012). Liposomes loaded with paclitaxel and modified with novel triphenylphosphonium-PEG-PE conjugate possess low toxicity, target mitochondria and demonstrate enhanced antitumor effects in vitro and in vivo. J. Control. Release.

[16] Yuan H.S., Cho H., Chen H.H., Panagia M., Sosnovik D.E., Josephson L. (2013). Fluorescent and radiolabeled triphenylphosphonium probes for imaging mitochondria. Chem. Commun..

[17] Hu Q.L., Gao M., Feng G.X., Liu B. (2014). Mitochondria-targeted cancer therapy using a light-up probe with aggregation-induced-emission characteristics. Angew. Chem. Int. Ed..

[18] Wang X.H., Peng H.S., Yang W., Ren Z.D., Liu Y.A. (2016). Mitochondria-targeted theranostic nanoparticles for optical sensing of oxygen, photodynamic cancer therapy, and assessment of therapeutic efficacy. Microchim. Acta.

[19] Lv W., Zhang Z., Zhang K.Y., Yang H.R., Liu S.J., Xu A.Q., Guo S., Zhao Q., Huang W. (2016). A mitochondria-targeted photosensitizer showing improved photodynamic therapy effects under hypoxia. Angew. Chem. Int. Ed..

[20] Chen X.W., Fu C.H., Wang Y.Q., Wu Q.R., Meng X.W., Xu K. (2018). Mitochondria-targeting nanoparticles for enhanced microwave ablation of cancer. Nanoscale.

[21] Kou J.Y., Dou D., Yang L.M. (2017). Porphyrin photosensitizers in photodynamic therapy and its applications. Oncotarget.

[22] Inada N.M., da Silva A.R., Jorge R.A., Borecky J., Vercesi A.E. (2007). Irradiated cationic mesoporphyrin induces larger damage to isolated rat liver mitochondria than the anionic form. Arch. Biochem. Biophys..

[23] Lei W.H., Xie J.F., Hou Y.J., Jiang G.Y., Zhang H.Y., Wang P.F., Wang X.S., Zhang B.W. (2010). Mitochondria-targeting properties and photodynamic activities of porphyrin derivatives bearing cationic pendant. J. Photochem. Photobiol. B.

[24] Rajaputra P., Nkepang G., Watley R., You Y. (2013). Synthesis and in vitro biological evaluation of lipophilic cation conjugated photosensitizers for targeting mitochondria. Bioorg. Med. Chem..

[25] Sharman W.M., Allen C.M., van Lier J.E. (1999). Photodynamic therapeutics: Basic principles and clinical applications. Drug Discov. Today.

[26] Ngen E.J., Rajaputra P., You Y. (2009). Evaluation of delocalized lipophilic cationic dyes as delivery vehicles for photosensitizers to mitochondria. Bioorg. Med. Chem..

[27] Cheng S.H., Lee C.H., Yang C.S., Tseng F.G., Mou C.Y., Lo L.W. (2009). Mesoporous silica nanoparticles functionalized with an oxygen-sensing probe for cell photodynamic therapy: Potential cancer theranostics. J. Mater. Chem..

[28] Detty M.R., Gibson S.L., Wagner S.J. (2004). Current clinical and preclinical photosensitizers for use in photodynamic therapy. J. Med. Chem..

[29] Guo F., Yu M., Wang J.P., Tan F.P., Li N. (2016). The mitochondria-targeted and IR780-regulated theranosomes for imaging and enhanced photodynamic/photothermal therapy. RSC Adv..

[30] Xiang H.J., Xue F.F., Yi T., Tham H.P., Liu J.G., Zhao Y.L. (2018). Cu_2–x_S nanocrystals cross-linked with chlorin e6-functionalized polyethylenimine for synergistic photodynamic and photothermal therapy of cancer. ACS Appl. Mater. Interfaces.

[31] Bernd A. (2014). Visible light and/or UVA offer a strong amplification of the anti-tumor effect of curcumin. Phytochem. Rev..

[32] Verwanger T., Krammer B., Bernardinelli E. (2011). Curcumin as a photosensitizer: Studies on different cell lines. Photodiagnosis Photodyn. Ther..

[33] Aggarwal B.B., Sundaram C., Mosley C.A., Liotta D.C., Menon V.P., Sudheer A.R., Shishodia S., Singh T., Surh Y.J., Chun K.S. (2007). The Molecular Targets and Therapeutic Uses of Curcumin in Health and Disease.

[34] Banik B., Somyajit K., Nagaraju G., Chakravarty A.R. (2014). Oxovanadium(IV) complexes of curcumin for cellular imaging and mitochondria targeted photocytotoxicity. Dalton Trans..

[35] Feng G.X., Liu B. (2016). Multifunctional AIEgens for future theranostics. Small.

[36] Hu F., Huang Y.Y., Zhang G.X., Zhao R., Yang H., Zhang D.Q. (2014). Targeted bioimaging and photodynamic therapy of cancer cells with an activatable red fluorescent bioprobe. Anal. Chem..

[37] Yuan Y.Y., Zhang C.J., Gao M., Zhang R.Y., Tang B.Z., Liu B. (2015). Specific light-up bioprobe with aggregation-induced emission and activatable photoactivity for the targeted and image-guided photodynamic ablation of cancer cells. Angew. Chem. Int. Ed..

[38] Yuan Y., Feng G., Qin W., Tang B.Z., Liu B. (2014). Targeted and image-guided photodynamic cancer therapy based on organic nanoparticles with aggregation-induced emission characteristics. Chem. Commun..

[39] Zhang C.J., Hu Q., Feng G., Zhang R., Yuan Y., Lu X., Liu B. (2015). Image-guided combination chemotherapy and photodynamic therapy using a mitochondria-targeted molecular probe with aggregation-induced emission characteristics. Chem. Sci..

[40] Guan Y., Lu H.G., Li W., Zheng Y.D., Jiang Z., Zou J.L., Gao H. (2017). Near-infrared triggered upconversion polymeric nanoparticles based on aggregation-induced emission and mitochondria targeting for photodynamic cancer therapy. ACS Appl. Mater. Interfaces.

[41] Yu C.Y.Y., Xu H., Ji S.L., Kwok R.T.K., Lam J.W.Y., Li X.L., Krishnan S., Ding D., Tang B.Z. (2017). Mitochondrion-anchoring photosensitizer with aggregation-induced emission characteristics synergistically boosts the radiosensitivity of cancer cells to ionizing radiation. Adv. Mater..

[42] Situ B., Chen S., Zhao E., Leung C.W.T., Chen Y., Hong Y., Lam J.W.Y., Wen Z., Liu W., Zhang W. (2016). Real-time imaging of cell behaviors in living organisms by a mitochondria-targeting AIE fluorogen. Adv. Funct. Mater..

[43] Zhao E., Deng H., Chen S., Hong Y., Leung C.W.T., Lam J.W.Y., Tang B.Z. (2014). A dual functional AEE fluorogen as a mitochondrial-specific bioprobe and an effective photosensitizer for photodynamic therapy. Chem. Commun..

[44] Gui C., Zhao E., Kwok R.T.K., Leung A.C.S., Lam J.W.Y., Jiang M., Deng H., Cai Y., Zhang W., Su H. (2017). AIE-active theranostic system: Selective staining and killing of cancer cells. Chem. Sci..

[45] Jiang M., Kwok R.T.K., Li X., Gui C., Lam J.W.Y., Qu J., Tang B.Z. (2018). A simple mitochondrial targeting AIEgen for image-guided two-photon excited photodynamic therapy. J. Mater. Chem. B.

[46] Liu J.P., Jin C.Z., Yuan B., Liu X.G., Chen Y., Ji L.N., Chao H. (2017). Selectively lighting up two-photon photodynamic activity in mitochondria with AIE-active iridium(III) complexes. Chem. Commun..

[47] Feng G.X., Liu J., Zhang C.J., Liu B. (2018). Artemisinin and AIEgen conjugate for mitochondria-targeted and image-guided chemo- and photodynamic cancer cell ablation. ACS Appl. Mater. Interfaces.

[48] Liu J.P., Chen Y., Li G.Y., Zhang P.Y., Jin C.Z., Zeng L.L., Ji L.N., Chao H. (2015). Ruthenium(II) polypyridyl complexes as mitochondria-targeted two-photon photodynamic anticancer agents. Biomaterials.

[49] Chakrabortty S., Agrawalla B.K., Stumper A., Vegi N.M., Fischer S., Reichardt C., Kögler M., Dietzek B., Feuring-Buske M., Buske C. (2017). Mitochondria targeted protein-ruthenium photosensitizer for efficient photodynamic applications. J. Am. Chem. Soc..

[50] Guo M., Xiang H.J., Wang Y., Zhang Q.L., An L., Yang S.P., Ma Y., Wang Y., Liu J.G. (2017). Ruthenium nitrosyl functionalized graphene quantum dots as an efficient nanoplatform for NIR-light-controlled and mitochondria-targeted delivery of nitric oxide combined with photothermal therapy. Chem. Commun..

[51] Chen S., Lei Q., Qiu W.X., Liu L.H., Zheng D.W., Fan J.X., Rong L., Sun Y.X., Zhang X.Z. (2017). Mitochondria-targeting “Nanoheater” for enhanced photothermal/chemo-therapy. Biomaterials.

[52] Marrache S., Dhar S. (2015). The energy blocker inside the power house: Mitochondria targeted delivery of 3-bromopyruvate. Chem. Sci..

[53] Ma Z.Y., Han K., Dai X.X., Han H.Y. (2018). Precisely striking tumors without adjacent normal tissue damage via mitochondria-templated accumulation. ACS Nano.

[54] Jung H.S., Han J., Lee J.H., Lee J.H., Choi J.M., Kweon H.S., Han J.H., Kim J.H., Byun K.M., Jung J.H. (2015). Enhanced NIR radiation-triggered hyperthermia by mitochondrial targeting. J. Am. Chem. Soc..

[55] Hou Z.Y., Zhang Y.X., Deng K.R., Chen Y.Y., Li X.J., Deng X.R., Cheng Z.Y., Lian H.Z., Li C.X., Lin J. (2015). UV-emitting upconversion-based TiO_2_ photosensitizing nanoplatform: Near-infrared light mediated in vivo photodynamic therapy via mitochondria-involved apoptosis pathway. ACS Nano.

[56] Yu Z.Z., Sun Q.Q., Pan W., Li N., Tang B. (2015). A near-infrared triggered nanophotosensitizer inducing domino effect on mitochondrial reactive oxygen species burst for cancer therapy. ACS Nano.

[57] Mou J., Lin T.Q., Huang F.Q., Shi J.L., Chen H.R. (2017). A new green titania with enhanced NIR absorption for mitochondria-targeted cancer therapy. Theranostics.

[58] Ma Z.F., Zhang M.C., Jia X.D., Bai J., Ruan Y.D., Wang C., Sun X.P., Jiang X.E. (2016). Fe^III^-doped two-dimensional C_3_N_4_ nanofusiform: A new O_2_-evolving and mitochondria-targeting photodynamic agent for MRI and enhanced antitumor therapy. Small.

[59] Wu H., Zeng F., Zhang H., Xu J.S., Qiu J.R., Wu S.Z. (2016). A nanosystem capable of releasing a photosensitizer bioprecursor under two-photon irradiation for photodynamic therapy. Adv. Sci..

[60] Tu Z.X., Qiao H.S., Yan Y.T., Guday G., Chen W., Adeli M., Haag R. (2018). Directed graphene-based nanoplatforms for hyperthermia: Overcoming multiple drug resistance. Angew. Chem. Int. Ed..

[61] Zhou H.Q., Fu C.H., Chen X.W., Tan L.F., Yu J., Wu Q., Su L.H., Huang Z.B., Cao F., Ren X.L. (2018). Mitochondria-targeted zirconium metal-organic frameworks for enhancing the efficacy of microwave thermal therapy against tumors. Biomater. Sci..

[62] Porcu E.P., Salis A., Gavini E., Rassu G., Maestri M., Giunchedi P. (2016). Indocyanine green delivery systems for tumour detection and treatments. Biotechnol. Adv..

[63] Luo S.L., Tan X., Fang S.T., Wang Y., Yuan Y., Sun H.Q., Qi Q.R., Shi C.M. (2016). Mitochondria-targeted small-molecule fluorophores for dual modal cancer phototherapy. Adv. Funct. Mater..

[64] Guo Q.Y., Luo S.L., Qi Q.R., Shi C.M. (2013). Preliminary structure-activity relationship study of heptamethine indocyanine dyes for tumor-targeted imaging. J. Innov. Opt. Health Sci..

[65] Lim S.Y., Hong K.H., Kim D.I., Kwon H., Kim H.J. (2014). Tunable heptamethine-azo dye conjugate as an NIR fluorescent probe for the selective detection of mitochondrial glutathione over cysteine and homocysteine. J. Am. Chem. Soc..

[66] Delaey E., van Laar F., De Vos D., Kamuhabwa A., Jacobs P., de Witte P. (2000). A comparative study of the photosensitizing characteristics of some cyanine dyes. J. Photochem. Photobiol. B.

[67] Kassab K. (2002). Photophysical and photosensitizing properties of selected cyanines. J. Photochem. Photobiol. B.

[68] Tan X., Luo S.L., Wang D.C., Su Y.P., Cheng T.M., Shi C.M. (2012). A NIR heptamethine dye with intrinsic cancer targeting, imaging and photosensitizing properties. Biomaterials.

[69] Luo S.L., Tan X., Qi Q.R., Guo Q.Y., Ran X.Z., Zhang L.L., Zhang E.L., Liang Y.F., Weng L.L., Zheng H. (2013). A multifunctional heptamethine near-infrared dye for cancer theranosis. Biomaterials.

[70] Thomas A.P., Palanikumar L., Jeena M.T., Kim K., Ryu J.H. (2017). Cancer-mitochondria-targeted photodynamic therapy with supramolecular assembly of HA and a water soluble NIR cyanine dye. Chem. Sci..

[71] Jung H.S., Lee J.H., Kim K., Koo S., Verwilst P., Sessler J.L., Kang C., Kim J.S. (2017). A mitochondria-targeted cryptocyanine-based photothermogenic photosensitizer. J. Am. Chem. Soc..

[72] Pan G.Y., Jia H.R., Zhu Y.X., Wang R.H., Wu F.G., Chen Z. (2017). Dual channel activatable cyanine dye for mitochondrial imaging and mitochondria-targeted cancer theranostics. ACS Biomater. Sci. Eng..

[73] Pan G.Y., Jia H.R., Zhu Y.X., Sun W., Cheng X.T., Wu F.G. (2018). Cyanine-containing polymeric nanoparticles with imaging/therapy-switchable capability for mitochondria-targeted cancer theranostics. ACS Appl. Nano Mater..

[74] Pan G.Y., Jia H.R., Zhu Y.X., Wu F.G. (2018). Turning double hydrophilic into amphiphilic: IR825-conjugated polymeric nanomicelles for near-infrared fluorescence imaging-guided photothermal cancer therapy. Nanoscale.

[75] Tan X., Luo S., Long L., Wang Y., Wang D., Fang S., Ouyang Q., Su Y.P., Cheng T.M., Shi C.M. (2017). Structure-guided design and synthesis of a mitochondria-targeting near-infrared fluorophore with multimodal therapeutic activities. Adv. Mater..

[76] Noh I., Lee D., Kim H., Jeong C.U., Lee Y., Ahn J.O., Hyun H., Park J.H., Kim Y.C. (2018). Enhanced photodynamic cancer treatment by mitochondria-targeting and brominated near-infrared fluorophores. Adv. Sci..

[77] Ge Y.L., Weng X.C., Tian T., Ding F., Huang R., Yuan L., Wu J., Wang T.L., Guo P., Zhou X. (2013). A mitochondria-targeted zinc(II) phthalocyanine for photodynamic therapy. RSC Adv..

[78] Marrache S., Tundup S., Harn D.A., Dhar S. (2013). Ex vivo programming of dendritic cells by mitochondria-targeted nanoparticles to produce interferon-gamma for cancer immunotherapy. ACS Nano.

[79] Yue C., Yang Y., Zhang C., Alfranca G., Cheng S., Ma L., Liu Y., Zhi X., Ni J., Jiang W. (2016). ROS-responsive mitochondria-targeting blended nanoparticles: Chemo- and photodynamic synergistic therapy for lung cancer with on-demand drug release upon irradiation with a single light source. Theranostics.

[80] Chennoufi R., Bougherara H., Gagey-Eilstein N., Dumat B., Henry E., Subra F., Bury-Moné S., Mahuteau-Betzer F., Tauc P., Teulade-Fichou M.P., Deprez E. (2016). Mitochondria-targeted triphenylamine derivatives activatable by two-photon excitation for triggering and imaging cell apoptosis. Sci. Rep..

[81] Liu H.W., Hu X.X., Li K., Liu Y.C., Rong Q.M., Zhu L.M., Yuan L., Qu F.L., Zhang X.B., Tan W.H. (2017). A mitochondrial-targeted prodrug for NIR imaging guided and synergetic NIR photodynamic-chemo cancer therapy. Chem. Sci..

[82] Pereira G.C., Branco A.F., Matos J.A., Pereira S.L., Parke D., Perkins E.L., Serafim T.L., Sardão V.A., Santos M.S., Moreno A.J. (2007). Mitochondrially targeted effects of berberine [Natural Yellow 18, 5,6-dihydro-9,10-dimethoxybenzo(g)-1,3-benzodioxolo(5,6-a) quinolizinium] on K1735-M2 mouse melanoma cells: Comparison with direct effects on isolated mitochondrial fractions. J. Pharmacol. Exp. Ther..

[83] Li Y., Tan C.P., Zhang W., He L., Ji L.N., Mao Z.W. (2015). Phosphorescent iridium(III)-bis-N-heterocyclic carbene complexes as mitochondria-targeted theranostic and photodynamic anticancer agents. Biomaterials.

[84] Cao J.J., Tan C.P., Chen M.H., Wu N., Yao D.Y., Liu X.G., Ji L.N., Mao Z.W. (2017). Targeting cancer cell metabolism with mitochondria-immobilized phosphorescent cyclometalated iridium(III) complexes. Chem. Sci..

[85] Li S.P.Y., Lau C.T.S., Louie M.W., Lam Y.W., Cheng S.H., Lo K.K.W. (2013). Mitochondria-targeting cyclometalated iridium(III)–PEG complexes with tunable photodynamic activity. Biomaterials.

[86] Reungpatthanaphong P., Dechsupa S., Meesungnoen J., Loetchutinat C., Mankhetkorn S. (2003). Rhodamine B as a mitochondrial probe for measurement and monitoring of mitochondrial membrane potential in drug-sensitive and -resistant cells. J. Biochem. Biophys. Methods.

[87] Wang Z., Chen Y.Z., Zhang H., Li Y.W., Ma Y.F., Huang J., Liu X.L., Liu F., Wang T.X., Zhang X. (2018). Mitochondria-targeting polydopamine nanocomposites as chemophotothermal therapeutics for cancer. Bioconjugate Chem..

[88] Chinnery P.F., Taylor R.W., Diekert K., Lill R., Turnbull D.M., Lightowlers R.N. (1999). Peptide nucleic acid delivery to human mitochondria. Gene Ther..

[89] Muratovska A., Lightowlers R.N., Taylor R.W., Wilce J.A., Murphy M.P. (2001). Targeting large molecules to mitochondria. Adv. Drug Deliv. Rev..

[90] Flierl A., Jackson C., Cottrell B., Murdock D., Seibel P., Wallace D.C. (2003). Targeted delivery of DNA to the mitochondrial compartment via import sequence-conjugated peptide nucleic acid. Mol. Ther..

[91] Fink M.P., Macias C.A., Xiao J., Tyurina Y.Y., Delude R.L., Greenberger J.S., Kagan V.E., Wipf P. (2007). Hemigramicidin-TEMPO conjugates: Novel mitochondria-targeted antioxidants. Crit. Care Med..

[92] Liu X.Y., Braun G.B., Zhong H.Z., Hall D.J., Han W.L., Qin M.D., Zhao C.Z., Wang M.N., She Z.G., Cao C.B. (2016). Tumor-targeted multimodal optical imaging with versatile cadmium-free quantum dots. Adv. Funct. Mater..

[93] Sibrian-Vazquez M., Nesterova I.V., Jensen T.J., Vicente M.G. (2008). Mitochondria targeting by guanidine– and biguanidine–porphyrin photosensitizers. Bioconjugate Chem..

[94] Han K., Lei Q., Wang S.B., Hu J.J., Qiu W.X., Zhu J.Y., Yin W.N., Luo X., Zhang X.Z. (2015). Dual-stage-light-guided tumor inhibition by mitochondria-targeted photodynamic therapy. Adv. Funct. Mater..

[95] Han K., Zhu J.Y., Jia H.Z., Wang S.B., Li S.Y., Zhang X.Z., Han H.Y. (2016). Mitochondria-targeted chimeric peptide for trinitarian overcoming of drug resistance. ACS Appl. Mater. Interfaces.

[96] Liu J.J., Liang H.N., Li M.H., Luo Z., Zhang J.X., Guo X.M., Cai K.Y. (2018). Tumor acidity activating multifunctional nanoplatform for NIR-mediated multiple enhanced photodynamic and photothermal tumor therapy. Biomaterials.

[97] Lu P., Bruno B.J., Rabenau M., Lim C.S. (2016). Delivery of drugs and macromolecules to the mitochondria for cancer therapy. J. Control. Release.

[98] Schleiff E., Becker T. (2011). Common ground for protein translocation: Access control for mitochondria and chloroplasts. Nat. Rev. Mol. Cell Biol..

[99] Stewart K.M. (2011). Design, synthesis, and characterization of a novel class of mitochondrial delivery vectors: Mitochondria-penetrating peptides. Ph.D. Thesis.

[100] Rizzuto R., Simpson A.W., Brini M., Pozzan T. (1992). Rapid changes of mitochondrial Ca^2+^ revealed by specifically targeted recombinant aequorin. Nature.

[101] Sibrian-Vazquez M., Jensen T.J., Hammer R.P., Vicente M.G.H. (2006). Peptide-mediated cell transport of water soluble porphyrin conjugates. J. Med. Chem..

[102] Sibrian-Vazquez M., Jensen T.J., Fronczek F.R., Hammer R.P., Vicente M.G.H. (2005). Synthesis and characterization of positively charged porphyrin−peptide conjugates. Bioconjugate Chem..

[103] Sibrian-Vazquez M., Jensen T.J., Vicente M.G. (2008). Synthesis, characterization, and metabolic stability of porphyrin−peptide conjugates bearing bifunctional signaling sequences. J. Med. Chem..

[104] Choi J., Shin J., Lee J., Cha M. (2012). Magnetic response of mitochondria-targeted cancer cells with bacterial magnetic nanoparticles. Chem. Commun..

[105] Ju E.G., Li Z.H., Liu Z., Ren J.S., Qu X.G. (2014). Near-infrared light-triggered drug-delivery vehicle for mitochondria-targeted chemo-photothermal therapy. ACS Appl. Mater. Interfaces.

[106] Gao G., Jiang Y.W., Jia H.R., Yang J.J., Wu F.G. (2018). On-off-on fluorescent nanosensor for Fe^3+^ detection and cancer/normal cell differentiation via silicon-doped carbon quantum dots. Carbon.

[107] Zhou F., Xing D., Wu B., Wu S., Ou Z., Chen W.R. (2010). New insights of transmembranal mechanism and subcellular localization of noncovalently modified single-walled carbon nanotubes. Nano Lett..

[108] Wei Y.C., Zhou F.F., Zhang D., Chen Q., Xing D. (2016). A graphene oxide based smart drug delivery system for tumor mitochondria-targeting photodynamic therapy. Nanoscale.

[109] Yang J.J., Zhang X.D., Ma Y.H., Gao G., Chen X.K., Jia H.R., Li Y.H., Chen Z., Wu F.G. (2016). Carbon dot-based platform for simultaneous bacterial distinguishment and antibacterial applications. ACS Appl. Mater. Interfaces.

[110] Yang J.J., Gao G., Zhang X.D., Ma Y.H., Jia H.R., Jiang Y.W., Wang Z.F., Wu F.G. (2017). Ultrasmall and photostable nanotheranostic agents based on carbon quantum dots passivated with polyamine-containing organosilane molecules. Nanoscale.

[111] Hua X.W., Bao Y.W., Chen Z., Wu F.G. (2017). Carbon quantum dots with intrinsic mitochondrial targeting ability for mitochondria-based theranostics. Nanoscale.

[112] Zhou F.F., Wu S.N., Wu B.Y., Chen W.R., Xing D. (2011). Mitochondria-targeting single-walled carbon nanotubes for cancer photothermal therapy. Small.

[113] Zhu Y.X., Jia H.R., Chen Z., Wu F.G. (2017). Photosensitizer (PS)/polyhedral oligomeric silsesquioxane (POSS)-crosslinked nanohybrids for enhanced imaging-guided photodynamic cancer therapy. Nanoscale.

[114] Bao Y.W., Hua X.W., Chen X.K., Wu F.G. (2018). Platinum-doped carbon nanoparticles inhibit cancer cell migration under mild laser irradiation: Multi-organelle-targeted photothermal therapy. Biomaterials.

[115] Wang H., Gao Z., Liu X.Y., Agarwal P., Zhao S.T., Conroy D.W., Ji G., Yu J.H., Jaroniec C.P., Liu Z.G. (2018). Targeted production of reactive oxygen species in mitochondria to overcome cancer drug resistance. Nat. Commun..

[116] Marrache S., Tundup S., Harn D.A., Dhar S. (2015). Ex vivo generation of functional immune cells by mitochondria-targeted photosensitization of cancer cells. Methods Mol. Biol..

[117] Yang G.B., Xu L.G., Xu J., Zhang R., Song G.S., Chao Y., Feng L.Z., Han F.X., Dong Z.L., Li B. (2018). Smart nanoreactors for pH-responsive tumor homing, mitochondria-targeting, and enhanced photodynamic-immunotherapy of cancer. Nano Lett..

[118] Jain M.B.S., Coulter J.A., Hounsell A.R., Butterworth K.T., McMahon S.J., Hyland W.B., Muir M.F., Dickson G.R., Prise K.M., Currell F.J. (2011). Cell-specific radiosensitization by gold nanoparticles at megavoltage radiation energies. Int. J. Radiat. Oncol. Biol. Phys..

[119] Werner M.E., Cummings N.D., Sethi M., Wang E.C., Sukumar R., Moore D.T., Wang A.Z. (2013). Preclinical evaluation of Genexol-PM, a nanoparticle formulation of paclitaxel, as a novel radiosensitizer for the treatment of non-small cell lung cancer. Int. J. Radiat. Oncol. Biol. Phys..

